# An engineered fatty acid synthase combined with a carboxylic acid reductase enables de novo production of 1-octanol in *Saccharomyces cerevisiae*

**DOI:** 10.1186/s13068-018-1149-1

**Published:** 2018-06-01

**Authors:** Sandra Henritzi, Manuel Fischer, Martin Grininger, Mislav Oreb, Eckhard Boles

**Affiliations:** 1 Faculty of Biological Sciences, Institute of Molecular Bioscience, Goethe University Frankfurt, Max-von-Laue-Str. 9, 60438 Frankfurt, Germany; 2 Institute of Organic Chemistry and Chemical Biology, Buchmann Institute for Molecular Life Sciences, Cluster of Excellence “Macromolecular Complexes”, Goethe University Frankfurt, Max-von-Laue-Str. 15, 60438 Frankfurt, Germany

**Keywords:** Fatty alcohol, 1-octanol, Carboxylic acid reductase, Biofuel, Octanoic acid, Caprylic acid, Fatty acid synthase, Short-chain fatty acids, Yeast, *Saccharomyces cerevisiae*

## Abstract

**Background:**

The ideal biofuel should not only be a regenerative fuel from renewable feedstocks, but should also be compatible with the existing fuel distribution infrastructure and with normal car engines. As the so-called drop-in biofuel, the fatty alcohol 1-octanol has been described as a valuable substitute for diesel and jet fuels and has already been produced fermentatively from sugars in small amounts with engineered bacteria via reduction of thioesterase-mediated premature release of octanoic acid from fatty acid synthase or via a reversal of the β-oxidation pathway.

**Results:**

The previously engineered short-chain acyl-CoA producing yeast Fas1^R1834K^/Fas2 fatty acid synthase variant was expressed together with carboxylic acid reductase from *Mycobacterium marinum* and phosphopantetheinyl transferase Sfp from *Bacillus subtilis* in a *Saccharomyces cerevisiae Δfas1 Δfas2 Δfaa2* mutant strain. With the involvement of endogenous thioesterases, alcohol dehydrogenases, and aldehyde reductases, the synthesized octanoyl-CoA was converted to 1-octanol up to a titer of 26.0 mg L^−1^ in a 72-h fermentation. The additional accumulation of 90 mg L^−1^ octanoic acid in the medium indicated a bottleneck in 1-octanol production. When octanoic acid was supplied externally to the yeast cells, it could be efficiently converted to 1-octanol indicating that re-uptake of octanoic acid across the plasma membrane is not limiting. Additional overexpression of aldehyde reductase Ahr from *Escherichia coli* nearly completely prevented accumulation of octanoic acid and increased 1-octanol titers up to 49.5 mg L^−1^. However, in growth tests concentrations even lower than 50.0 mg L^−1^ turned out to be inhibitory to yeast growth. In situ extraction in a two-phase fermentation with dodecane as second phase did not improve growth, indicating that 1-octanol acts inhibitive before secretion. Furthermore, 1-octanol production was even reduced, which results from extraction of the intermediate octanoic acid to the organic phase, preventing its re-uptake.

**Conclusions:**

By providing chain length control via an engineered octanoyl-CoA producing fatty acid synthase, we were able to specifically produce 1-octanol with *S. cerevisiae*. Before metabolic engineering can be used to further increase product titers and yields, strategies must be developed that cope with the toxic effects of 1-octanol on the yeast cells.

## Background

Dwindling fossil resources and a growing global energy demand, especially in the sector of human mobility and transportation, are leading to economic and environmental burdens. This development poses a serious threat for the environment with respect to emissions of greenhouse gases and particulate matter from traditional fuels like gasoline and diesel [1, 2]. An alternative is the development of sustainable and regenerative fuels from renewable feedstocks. However, those substitutes are not always compatible with the existing infrastructure for distribution or with traditional vehicle engines [3], but may require technical modifications of engines due to different physicochemical properties and combustion behaviors. Therefore, current research focuses on the application of so-called *drop*-*in* biofuels. They are considered as complete replacements of fossil fuels or as additives for blending due to similar characteristics regarding critical parameters [4, 5]. Among a comprehensive portfolio of approved molecules from microbial production [5–9], 1-octanol has acquired special attention as substitute for diesel and jet fuels [10–13]. Previous studies [10, 14] compared various characteristics of fossil-derived as well as bio-derived diesel fuels with saturated short- and medium-chain alcohols, and showed that 1-octanol exhibits best matching overall properties compared to ethanol or other long-chain alcohols.

**Fig. 1:**
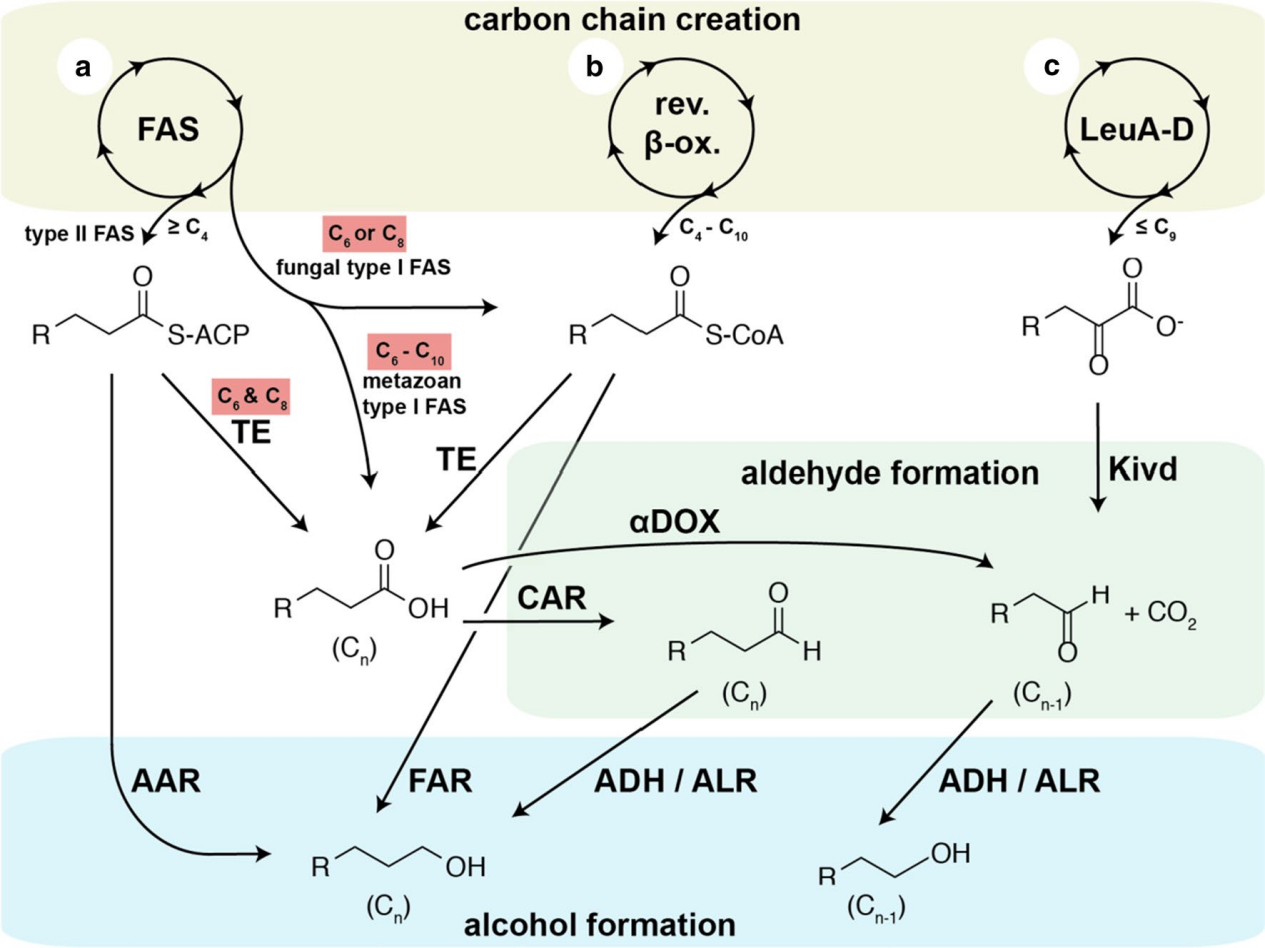
Common pathways for microbial higher alcohol production. Three different recursive metabolic pathways, fatty acid synthesis (**a**), reverse β-oxidation (**b**), or an artificial pathway engineered from l-leucine biosynthesis with enzymes LeuA to LeuD (**c**) can serve to provide different precursors with carbon chain lengths as indicated. Reaction steps for which shifted chain length control has successfully been reported are highlighted in red. For description and abbreviations, see text

Various approaches are under investigation for the microbial synthesis of higher unbranched alcohols with respect to the origin of the saturated carbon chain and the formation of the terminal hydroxyl group. They are based to some extent on entirely different metabolic pathways in bacterial as well as yeast systems (Fig. 1). Most approaches aim at harvesting acyl chain for the synthesis of the higher alcohol. Two major biochemical strategies can be distinguished for the acyl chain synthesis: (1) The alpha-ketoacid route [15] exerts the recursive elongation of alpha-ketoacids by one carbon atom with an adapted leucine biosynthesis pathway and gives access to saturated carbon chains from C_3_ to C_9_ [16]. (2) Fatty acid synthesis or an artificially induced reverse β-oxidation [17, 18] exerts two-carbon elongation of beta-ketoacids for saturated acyl chain creation.

In all these cases, the desired alcohol must be formed in a final reaction step via reduction of a corresponding precursor, either CoA-thioester, ACP-tethered thioester, or free carboxylate. Fatty acyl-CoA reductases (FAR) and acyl-ACP reductases (AAR) proved to be suitable for the reduction of both thioester species, which they directly reduce to the corresponding alcohol [17, 19–26] and often do not discriminate between ACP and CoA as carrier [27]. A free carboxylate is comparably unreactive, but can be efficiently converted to a C_n-1_ aldehyde by α-dioxygenases (αDOX) under decarboxylative elimination of the terminal carboxyl group [28] or to a C_n_ aldehyde by carboxylic acid reductases (CAR) [29–31]. The CAR-enzyme family requires a phosphopantetheinylation by a phosphopantetheinyl transferase to be active [32]. The aldehydes can be further reduced to alcohols by various aldehyde reductases (ALR) or alcohol dehydrogenases (ADH) [6, 33]. A broad applicability of all these reducing enzymes is facilitated by a promiscuous substrate acceptance of FARs/AARs [19, 20, 23, 34, 35] as well as CAR [31, 36, 37] and ALR/ADH [30]. In aiming for microbial short/medium-chain alcohol production, the bottleneck does therefore rather lie in the supply of the respective short/medium-chain acyl chain than the downstream reducing enzymes. This is reflected in the variety of publications about fatty alcohol production derived from the naturally defined pool of fatty acids in the C_16_/C_18_-range [23, 25, 38], while work on short/medium-chain alcohol production is rare. Marcheschi et al. [39] reported the production of mixed C_3_–C_8_-alcohols with the α–ketoacid route using mutated LeuA variants in *E. coli*, but with a maximal 1-octanol yield of 15 mg L^−1^, which accounts for only 0.1% in this mixture. Higher titers were reported using a reverse β-oxidation route and an aldehyde/alcohol dehydrogenase AdhE2 from *Clostridium acetobutylicum* for reducing the obtained CoA-ester to give 65 mg L^−1^ 1-octanol as one component of a C_2_–C_10_ alcohol mixture in *E. coli* [40] or even 100 mg L^−1^ as minor product after further metabolic optimizations [18]. For the direct usage of fatty acyl-CoAs as precursors, Sheng et al. [41] reported an elaborate strategy to escape from the normal product spectrum of mainly C_16_/C_18_. They directed a fatty acyl-CoA reductase to the peroxisome to capture medium-chain substrates from β-oxidation, which resulted in the production of 1 g L^−1^ C10, C_12_ and C_16_ fatty alcohols in *S. cerevisiae* [41]. In spite of the success of producing short/medium-chain alcohols, these examples illustrate the difficulty of chain length control that causes dissipation of synthetic capacity and finally requires separation of the desired 1-octanol from product mixtures later on. Akhtar et al. [14] were first in publishing the introduction of a thioesterase, Tes3 from *Anaerococcus tetradius,* in a CAR/Ahr-expressing *E. coli*, which is highly specific for C_6_- and C_8_-acyl thioesters. In this way, they harvested immature fatty acyl-ACPs from the bacterial fatty acid biosynthesis and obtained up to 62 mg L^−1^ 1-octanol besides 29 mg L^−1^ 1-hexanol with laboratory *E. coli* BL21 cells [14].

Yeast as the applied organism offers certain advantages over the hitherto exclusively reported *E. coli* systems for microbial 1-octanol production. *S. cerevisiae* is highly robust, shows a high tolerance to stress in fermentative processes, and ferments sugars at low pH values [42, 43]. Also, many genetic manipulation tools are established which make yeast a very attractive organism for metabolic engineering [44]. Several studies have been dealing with the overproduction of long-chain fatty acids and derivatives in *S. cerevisiae* [24–26, 30, 45].

Due to the spatial encapsulation of fatty acid synthesis in fungi in a barrel-shaped multienzyme complex [46–50], the strategy of hydrolyzing short/medium-chain acyl-ACP with specific thioesterases, as e.g., Tes3, is difficult to apply for this system. In previous studies, this was addressed by introducing a structurally different metazoan fatty acid synthase with fused thioesterase [51] or the incorporation of a thioesterase in the fungal fatty acid synthase’s reaction chamber [52]. We have recently reported the rational engineering of the type I fatty acid synthase (FAS) from *S. cerevisiae* for the selective production of short/medium-chain fatty acids (C_6_ or C_8_) [53] based on a preceding in vitro/in silico approach [54]. Mutations in the selected enzymatic domains, controlling fatty acid chain length, led to a total yield of 245 mg L^−1^ (C_6_ or C_8_ fatty acid) and a specificity for C_8_ fatty acids of 90% (of secreted fatty acids), resembling to date the most efficient and most specific short/medium-chain fatty acids producing yeast strains [51, 52, 55, 56]. We consider this strain as ideal platform to produce 1-octanol. Here, we show that combining an octanoyl-CoA producing engineered FAS from yeast with heterologously expressed carboxylic acid reductases and aldehyde reductases, together with endogenous thioesterases and alcohol dehydrogenases, enables efficient de novo production of 1-octanol from glucose with *S. cerevisiae*.

## Results and discussion

### Biosynthesis of 1-octanol from glucose

**Fig. 2:**
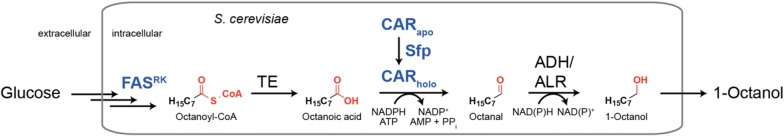
Metabolic pathway for 1-octanol production in *S. cerevisiae* from glucose via fatty acid biosynthesis. A mutant version of *S. cerevisiae* FAS (Fas1^R1834K^/Fas2) produces octanoyl-CoA which is hydrolyzed by endogenous thioesterases (TE) to the free octanoic acid [53]. A heterologous carboxylic acid reductase (CAR) from *M. marinum* then converts the free fatty acid to octanal which is further reduced to 1-octanol by endogenous alcohol dehydrogenases (ADH) and aldehyde reductases (ALR) [29]. CAR must be activated by the phosphopantetheinyl tranferase Sfp from *Bacillus subtilis* [31]. Heterologous or mutated enzymes are marked in blue. FAS^RK^ means FAS^R1834K^

In this study, we engineered a synthetic pathway to produce 1-octanol from glucose in *S. cerevisiae* in which the chain length of the fatty alcohol is determined by the product release of a mutated yeast FAS, namely Fas1^R1834K^/Fas2 [53]. Host strain RPY21 which exhibits deletions of both *FAS* genes, *FAS1* and *FAS2*, as well as the gene *FAA2* encoding a short/medium-chain fatty acyl-CoA synthetase, was transformed with centromeric vectors expressing *FAS1*^*R1834K*^ and *FAS2* under control of their native promoters. We had previously reported that the FAS^R1834K^ mutant version of FAS in concert with the thioesterase activities of three short-chain acyl-CoA:ethanol acyltransferases, Eht1, Eeb1, and Mgl2, produces high amounts of octanoic acid [53]. To avoid subsequent degradation of octanoic acid, we additionally deleted *FAA2* which is required for the re-activation of octanoic acid to octanoyl-CoA initiating its degradation by β-oxidation [57]. Interestingly, it has been reported before that the deletion of *FAA2* already in a wild-type FAS background leads to the production of low amounts of octanoic acid [58]. In the FAS^R1834K^ mutant background, the deletion of *FAA2* resulted in an increase of up to 25% in octanoic acid production (301 mg L^−1^; data not shown). To produce 1-octanol from free octanoic acid, we overexpressed a heterologous carboxylic acid reductase (CAR) from *Mycobacterium marinum* under the control of the strong and constitutive *HXT7*^−*1*-−*392*^ promotor fragment together with the phosphopantetheinyl transferase Sfp from *Bacillus subtilis* under the control of the strong *PGK1* promoter on a multicopy vector in RPY21 expressing Fas1^R1834K^/Fas2 [14]. CAR uses NADPH and ATP in order to reduce free fatty acids to the corresponding aldehydes [31] (Fig. 2) and must be activated by a phosphopantetheine transferase: in this study, Sfp, which attaches the prosthetic group 4′-phosphopantetheine to the enzyme [31, 32]. The aldehydes can be further reduced to primary alcohols by endogenous alcohol dehydrogenases (ADH) or aldehyde reductases (ALR) in yeast [29, 33].

**Fig. 3:**
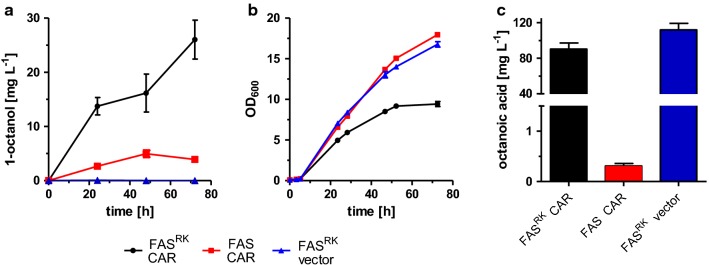
Production of 1-octanol with *S. cerevisiae.* Strain RPY21 expressing mutated FAS^R1834K^ as well as CAR and Sfp from a high-copy plasmid (black), was grown for 72 h in buffered YPD medium. For reference, analogous combinations of wild-type FAS, CAR, and Sfp (red) as well as mutated FAS^R1834K^ with empty vector instead of CAR and Sfp are shown (blue). Error bars reflect the standard deviations from three biological replicates. OD_600_ (**a**) and extracellular 1-octanol concentrations (**b**) were analyzed at different time points. **c** Final octanoic acid concentration in the extracellular culture medium after 72 h. FAS^RK^ means FAS^R1834K^

Cultivation of the yeast cells in potassium phosphate buffered medium at pH 6.5 with 2% glucose under aerobic conditions resulted in the accumulation of 1-octanol in the extracellular medium with titers of up to 26.0 ± 3.6 mg L^−1^ (2.8 ± 0.3 mg L^−1^ OD_600_^−1^) after 72 h (Fig. 3a), which was also indicated by a strong specific smell of the cell cultures. The production of 1-octanol confirmed that *S. cerevisiae* contains suitable endogenous ADHs/ALRs for the reduction of 1-octanal (Fig. 2) as shown earlier [29]. Besides 1-octanol, also small amounts of 1-hexanol (5.5 ± 0.6 mg L^−1^ after 72 h; data not shown) could be determined. This was expected since FAS^R1834K^ also produces small amounts of hexanoic acid which is then also reduced by CAR to its corresponding aldehyde [14, 31, 53]. In contrast, although decanoic acid was another side product of FAS^R1834K^ [53], decanol was not detected.

It is worthy to mention that even the control strain RPY21, expressing wild-type FAS and overexpressing CAR and Sfp, secreted 1-octanol in small amounts (3.9 ± 0.1 mg L^−1^) (Fig. 3a), which has also been reported before in a comparable setup [29]. Possibly, this is the result of the small amounts of octanoic acid produced by wild-type FAS or the mitochondrial type II FAS system in *S. cerevisiae* [53, 54, 59], especially together with deletion of *FAA2* as in strain RPY21 [58].

To determine the limiting factors in 1-octanol production in strain RPY21/FAS^R1834K^ overexpressing CAR and Sfp, we furthermore analyzed the accumulation of free octanoic acid in the culture medium, and detected a considerable titer of 90.3 ± 6.8 mg L^−1^ after 72 h (Fig. 3c). Compared to the same strain without CAR which accumulated 118.9 ± 7.3 mg L^−1^ octanoic acid, this reveals that 76% of precursor substrate remained unused for octanol production, suggesting a bottleneck either in the CAR or the ADH/ALR reactions, or losses due to secretion of octanoic acid out of the cells.

**Fig. 4:**
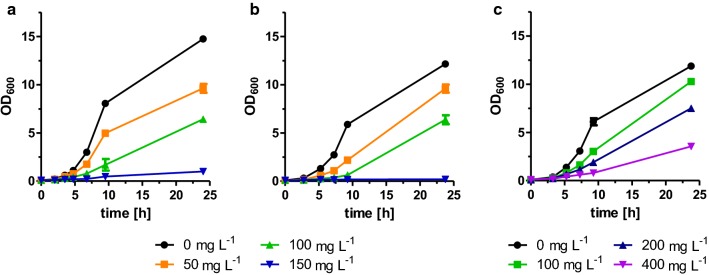
Inhibitory effects of 1-octanol, octanal, and octanoic acid added to the culture medium of strain BY4741. The yeast strain BY4741 was cultivated for 24 h in buffered YPD medium supplemented with different concentrations of **a** 1-octanol **b** octanal, or **c** octanoic acid. Error bars reflect the standard deviations from two biological replicates. OD_600_ was analyzed at different time points

The decreased maximal optical density after 72 h of the yeast culture RPY21/FAS^R1834K^ overexpressing CAR and Sfp indicated that 1-octanol or the other pathway intermediates octanoic acid and octanal might have a toxic effect on the yeast cells and inhibit their growth (Fig. 3b). To test the inhibitory effects, different concentrations of 1-octanol, octanal, and octanoic acid were added to yeast cultures of the wild-type strain BY4741 and growth curves were determined (Fig. 4). Indeed, at the lowest tested concentration of 50 mg L^−1^ of 1-octanol or octanal in the culture medium, slightly inhibited growth of the yeast cells was observed, and growth was completely prevented at a concentration of 150 mg L^−1^ (Fig. 4a, b). In contrast, octanoic acid is less toxic, and moderate cell growth was even observed at a concentration of 400 mg L^−1^ (Fig. 4c).

### Re-uptake of secreted octanoic acid from the medium

**Fig. 5:**
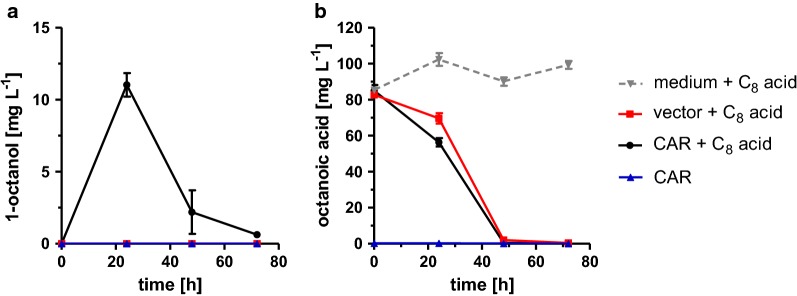
Uptake and conversion analysis of octanoic acid. **a** 1-Octanol production and **b** octanoic acid consumption of *S. cerevisiae* BY4741 expressing CAR and Sfp from a multicopy plasmid. Cultivation was performed for 72 h in buffered YPD medium without and with supplementation of 90 mg L^−1^ octanoic acid. For reference, noninoculated cultivation medium was treated analogously. Error bars reflect the standard deviations from three biological replicates

To rule out that the secretion of octanoic acid into the culture medium is a limiting factor for 1-octanol production, we analyzed the transport of octanoic acid back into the cell and its further conversion to 1-octanol by overexpressing CAR and Sfp in the wild-type strain BY4741 in buffered media supplemented with 90 mg L^−1^ octanoic acid. After 24 h, 11.0 ± 0.8 mg L^−1^ 1-octanol was detected (Fig. 5a), showing that octanoic acid can be transported back into the cell and subsequently be reduced by CAR together with ADHs/ALRs to 1-octanol. Nevertheless, the concentration of octanoic acid in the medium also decreased in the absence of a CAR (Fig. 5b), indicating alternative reaction pathways for octanoic acid conversion in *S. cerevisiae*. Probably, this is due to degradation of octanoic acid via β-oxidation in strain BY4741 [57, 60]. Moreover, the amount of 1-octanol in the extracellular medium also decreased after 24 h (Fig. 5a). This might be due to its reconversion to octanal by endogenous alcohol dehydrogenases which then might be oxidized, e.g., by the dehydrogenase Hfd1, which has been reported to convert at least long-chain aliphatic aldehydes to carboxylic acids [33].

### Overexpression of an aldehyde reductase Ahr from *E. coli* increases 1-octanol production

**Fig. 6:**
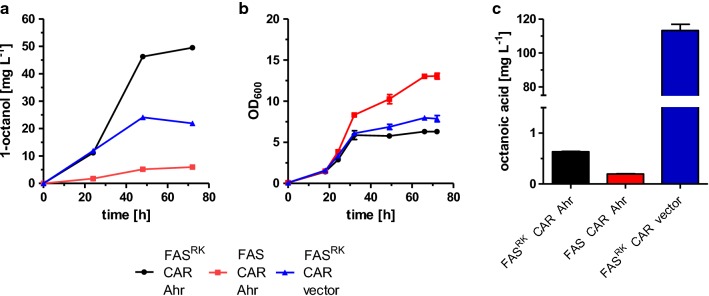
Increase in 1-octanol production in a yeast strain by additional overexpression of heterologous Ahr. Strain RPY21/FAS^R1834K^ expressing CAR and Sfp together with Ahr from *E. coli* from a high-copy plasmid (black), was grown for 72 h in buffered YPD medium. For comparison, analogous combinations of wild-type FAS with CAR, Sfp, and Ahr (red) as well as mutated FAS^R1834K^ with empty vector instead of Ahr are shown (blue). Error bars indicate the standard deviation from three biological replicates. **a** OD_600_ and 1-octanol **b** were analyzed at different time points. **c** Final octanoic acid concentration after 72 h. FAS^RK^ means FAS^R1834K^

The previous experiment showed that octanoic acid can freely diffuse or is transported in both directions across the yeast plasma membrane, and therefore should not be limiting 1-octanol production. Therefore, we tested octanal reduction as a limiting factor in 1-octanol production. For this reaction, *S. cerevisiae* contains various endogenous ADHs/ALRs, successfully employed for the aldehyde–alcohol conversion in the C_8_–C_18_ range already earlier [29]. However, little is known about the efficiencies of these enzymes to short/medium-chain fatty aldehydes, especially to octanal. It has been shown by Akhtar et al. [31] that the aldehyde reductase Ahr from *E. coli* accepts a broad range of aliphatic aldehydes (C_4_ to C_16_). We overexpressed Ahr from *E. coli* under the control of the *HXT7*^−*1*-−*392*^ promotor fragment from a high-copy plasmid together with the plasmid encoding for CAR and Sfp in strain RPY21/FAS^R1834K^ under aerobic conditions. Overexpression of Ahr led to a twofold increase in the absolute 1-octanol titer (49.5 ± 0.8 mg L^−1^ after 72 h) in the extracellular medium (Fig. 6a). This increase in 1-octanol production is also reflected in a decreased maximal optical density of the cell culture after 72 h compared to the control strain without Ahr (Fig. 6b), probably due to the negative effect of 1-octanol on cell growth (Fig. 4a). When the 1-octanol titers are normalized to the final OD_600_ of the cultures, overexpression of Ahr resulted in a threefold increase of 1-octanol (7.9 ± 0.4 mg L^−1^ OD_600_^−1^ compared to 2.8 ± 0.3 mg L^−1^ OD_600_^−1^ after 72 h). Furthermore, the presence of Ahr nearly completely prevented the accumulation of octanoic acid (0.6 ± 0.0 mg L^−1^ after 72 h) compared to the strain without Ahr (113.2 ± 2.6 mg L^−1^) (Fig. 6c). This result revealed that the reduction of octanal to 1-octanol by endogenous ADHs and ALRs was a limiting step in the original pathway.

### In situ extraction of 1-octanol

**Fig. 7:**
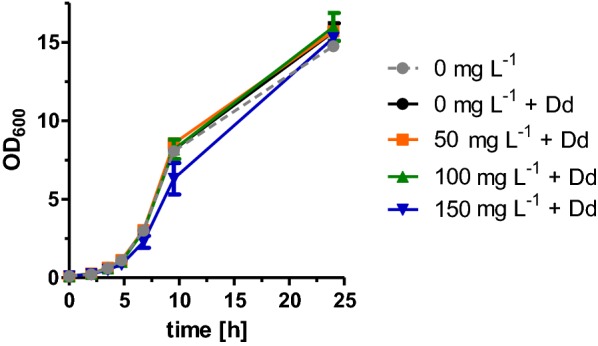
Growth behavior of strain BY4741 in the presence of 20% dodecane and different 1-octanol concentrations. BY4741 was cultivated up to 24 h in buffered YPD medium overlaid with 20% dodecane (Dd) and supplemented with different 1-octanol concentrations. For comparison, also the growth curve of a culture without dodecane was determined (gray). Error bars reflect the standard deviations from two biological replicates. OD_600_ was analyzed at different time points

**Fig. 8:**
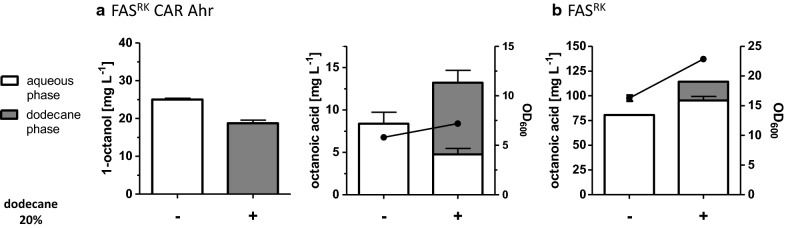
*In situ* extraction of 1-octanol and octanoic acid in a two-phase fermentation of yeast. Strain RPY21/FAS^R1834K^ overexpressing CAR and Sfp together with Ahr (**a**) and control strain RPY21/FAS^R1834K^ (**b**) were grown for 72 h, in buffered YPD medium overlaid with (+) and without (−) 20% of dodecane. Error bars reflect the standard deviations from two biological replicates. After 72 h, the final OD_600_ (·) and the final concentrations of 1-octanol and octanoic acid were analyzed. FAS^RK^ means FAS^R1834K^. The gray areas indicate concentrations of 1-octanol or octanoic acid, respectively, calculated on the basis of the volume of the aqueous phase of the culture, but based on the amounts measured in the dodecane phase. The white-colored areas indicate concentrations measured directly in the aqueous phase

Since already minor concentrations of 1-octanol inhibited growth of the yeast cells (Fig. 4a), which makes further improvements toward higher 1-octanol titers difficult, one possibility to circumvent this problem could be an in situ extraction of released 1-octanol in a two-phase fermentation using an organic solvent as the secondary phase. An appropriate solvent is dodecane which was shown to have no negative effects on yeast growth (Fig. 7) [61, 62] and was already used for production of long-chain fatty alcohols [22, 24]. To test if dodecane as secondary phase can improve growth in the presence of 1-octanol by trapping it out of the yeast culture, different concentrations of 1-octanol were added to yeast cultures of the wild-type strain BY4741 overlaid with 20% dodecane, and growth curves were determined (Fig. 7). Indeed, this time, the addition of even 150 mg L^−1^ 1-octanol to the yeast culture with 20% dodecane did not affect growth. Based on these results, a two-phase fermentation with the 1-octanol producing strain was performed using dodecane as a secondary phase for in situ extraction. To achieve this, we overexpressed Ahr, CAR, and Sfp from high-copy plasmids in strain RPY21/FAS^R1834K^ under aerobic conditions in buffered media overlaid with and without 20% of dodecane. After 72 h, the growth was determined by OD_600_ measurements, and the amounts of fatty acids and fatty alcohols in the aqueous and in the dodecane phase were analyzed. We found that in the two-phase fermentation, no 1-octanol was detectable in the aqueous phase revealing the efficient extraction of 1-octanol. However, analysis of 1-octanol in the dodecane layer showed that 1-octanol production was reduced by about 25% compared to the strain cultivated without dodecane (Fig. 8a) (it should be noted that the concentration of 1-octanol determined in the dodecane phase was calculated on the volume of the aqueous phase of the culture). This is likely due to the sequestration of octanoic acid into the dodecane phase (Fig. 8a). Cultivation of the strain RPY21/FAS^R1834K^ without the 1-octanol pathway (which yields higher titers of octanoic acid) in buffered medium overlaid with 20% dodecane confirmed the partial accumulation of octanoic acid in the organic phase (Fig. 8b). Although addition of dodecane could circumvent the inhibitory effect of added 1-octanol on growth (Fig. 7), surprisingly, this was not the case when 1-octanol was produced by the cells themselves (Fig. 8a). Here, the growth of the 1-octanol producing yeast cultures with dodecane was only slightly improved. Taken together, in situ extraction could not improve 1-octanol production and the speedup of the downstream pathway for octanoic acid production is necessary to compensate the loss of octanoic acid into the organic layer. The results also suggest that beside the toxic effect of 1-octanol, its production somehow also inhibits growth of the cells which might partially be connected to a limited supply of ATP and the cofactor NADPH required for octanoic acid production as well as for the CAR and Ahr reactions.

## Conclusion

This is the first study to report on dedicated production of 1-octanol in *S. cerevisiae*. We achieved the nearly selective production of 1-octanol in *S. cerevisiae* by combining a proprietary C_8_-acid producing FAS^R1834K^-mutant with a two-step reduction pathway composed of CAR, Sfp, and Ahr [14, 53]. In the process of the current study, the chain length specificity of the carbon chain-providing FAS is the decisive step to ensure specific 1-octanol production. Nevertheless, toxicity of 1-octanol and a negative effect of its production on growth of the cells pose fresh challenges for further optimizations.

## Methods

### Yeast strain, media, and transformation

The haploid *S. cerevisiae* strain RPY21 used in this study for fatty alcohol production has a BY background and is based on BY.PK1238_1A_KO with knocked out *FAS1* and *FAS2* from previous studies [53]. The deletion of the fatty acyl-CoA synthetase Δ*faa2* was generated using the CRISPR–Cas9 system [63]. The relevant genotype of RPY21 is *Matα; ura3Δ0; his3Δ0; leu2Δ0; TRP1; lys2Δ0; MET15; fas1::uptag*-*kanMX4*-*downtag; fas2::uptag*-*kanMX4*-*downtag*; *Δfaa2*. RPY21 was transformed as described in Gajewski et al. [53]. Selection of yeast transformants was done on the defined synthetic complete media (SCD) as described in Bruder et al. [64] without leucine and histidine containing the respective antibiotics (200 µg mL^−1^ hygromycin; 100 µg mL^−1^ nourseothricin sulfate). The strain BY4741 and the strain CEN.PK2-1C for plasmid construction by homologous recombination were transformed according to protocols by Gietz and Schiestl [65].

### Growth experiments

The strain BY4741 was pregrown in YPD medium buffered with 100 mM potassium phosphate and adjusted to a pH of 6.5 as described in Gajewski et al. [53] without supplementation of free FAs. After washing steps, the main culture (50 mL YPD in 300 mL flasks; two biological replicates) was inoculated to OD_600_ = 0.1, supplemented with different concentrations of 1-octanol (0, 50, 100, and 150 mg L^−1^), octanal (0, 50, 100, and 150 mg L^−1^), or octanoic acid (0, 100, 200, and 400 mg L^−1^) and aerobically shaken for 24 h at 180 r.p.m. at 30 °C. OD_600_ was analyzed at different time points.

### *S. cerevisiae* fermentations

*Saccharomyces cerevisiae* strains were cultured in YPD medium as described in Gajewski et al. [53] without supplementation of free FAs. The medium was additionally buffered with 100 mM potassium phosphate and adjusted to a pH of 6.5. To test the 1-octanol production of the different strains, a preculture was inoculated in buffered YPD medium and grown aerobically to exponential phase. After washing steps, the main culture (50 mL YPD with respective antibiotics; two to three biological replicates) was inoculated to OD_600_ = 0.1 and aerobically shaken for 72 h at 180 r.p.m. and 30 °C.

For the in situ extraction, the main culture (25 mL YPD with respective antibiotics; two biological replicates) was inoculated to OD_600_ = 0.1 and overlaid with 20% dodecane (5 mL). Fatty alcohols, fatty acids, and OD_600_ were analyzed at different time points. The final 1-octanol concentration after 72 h was normalized to the final OD_600_ value (mg L^−1^ OD_600_).

### Plasmid and strain construction

The plasmids used in this study are listed in Table 1. FAS-related plasmids are described in Gawjeski et al. [53]. Genes encoding carboxylic acid reductase *CAR* from *M. marinum (*UniProt: B2HN69*)* and the phosphopantetheinyl transferase *Sfp* from *B. subtilis* (UniProt: P39135) were codon-optimized according to the yeast glycolytic codon usage [66]. The plasmid pRS62H–CAR–Sfp was assembled by homologous recombination in yeast with four PCR fragments with 30 bp overlaps. Yeast was transformed as described above with a mixture of these fragments generated by PCR using primers shown in Table 2. The assembled plasmids were recovered by yeast DNA preparations and transformed into *E. coli* for amplification by standard procedures. The construction of the plasmid pRS62N-Ahr was performed by Gibson assembly as described in Gibson et al. [67]. The bacterial gene *Ahr* [previously known as *yigB* (UniProt: P27250)] was amplified from chromosomal DNA of *E. coli* DH5α using primers shown in Table 2.

**Table 1:** List of plasmids used in this study

Plasmid name	Description	References
pRS315-FAS1-Wt	*CEN6ARS4*, *AmpR*, *LEU2*, *p*_*FAS1*_-^*Sc*^*FAS1*-*Wt*-*t*_*FAS1*_	[53]
pRS315-FAS1-RK	*CEN6ARS4*, *AmpR, LEU2, p*_*FAS1*_-^*Sc*^*FAS1*^*R1834K*^-*t*_*FAS1*_	[53]
pRS313-FAS2-Wt	*CEN6ARS4*, *AmpR, HIS3*, *p*_*FAS2*_-^*Sc*^*FAS2*-*Wt*-*t*_*FAS2*_	[53]
pRS62H	*2µ*, *hphNT1*, *AmpR*, truncated *HXT7* promotor (p_HXT7_^−1-−392^) and *FBA1* terminator	[68]
pRS62 N	*2µ*, *natNT2, AmpR,* shortened *HXT7* promotor (p_HXT7_^−1-−392^) and *CYC1* terminator	[68]
pRS62H-CAR-Sfp	pRS62H; *p*_*HXT7*_^−*1*-−*392*^-^*Mm*^*CAR*-*t*_*FBA*_*; p*_*PGK1*_-^*Bs*^*Sfp*-*t*_*PGM2*_	This study
pRS62 N-Ahr	pRS62N; *p*_*HXT7*_^−*1*-−*392*^-^*Ec*^*Ahr*-*t*_*CYC1*_	This study

Genes from *Saccharomyces cerevisiae* (*Sc*), *Mycobacterium marinum* (*Mm*), *E. coli* (*Ec*), and *Bacillus subtilis* (*Bs*) are indicated by prefixes in superscript. *hphNT1* hygromycin resistance, *Amp* ampicillin resistance, *natNT2* nourseothricin sulfate resistance

**Table 2:** Relevant primers for plasmid construction

Primer name	Sequence 5′–3′	Amplicon
SHP35_pPGK1_for	AACCCTGGCGTTACCCAACTTAATCGCCTTGCAG CATGTTTGCAAAAAGAACAAAACTG	*PGK1* promotor
CBP160_pPGK1_rev	TGTTTTATATTTGTTGTAAAAAGTAGATAATTAC	*PGK1* promotor
CBP35_tPGM2_for	AACGAATGATTTACTAATGGC	*PGM2* terminator
SHP36_tPGM2_rev	GAAATCGGCAAAATCCCTTATAAATCAAAAGAAT AGACAAAAAACTCGGGGTAGGTAAT	*PGM2* terminator
SHP54_Ahr_for	CAAAAACAAAAAGTTTTTTTAATTTTAATCAAAAA ATGTCGATGATAAAAAGCTATGCC	*Ahr* ORF
SHP53_Ahr_rev	ATGTAAGCGTGACATAACTAATTACATGACTCGA GTCAAAAATCGGCTTTCAACAC	*Ahr* ORF
DR_Faa2	GGAAGAATGCAGGTTACAAAAAACGGATAAGAA CAAACTTGTTTCGAAATGTACTTATGACGATTTGG AACACATTCAAACTAGAAAAAACTTTGATGTA	Donor-DNA fragment for *FAA2* deletion
CC_FAA2-rev	CGTAAGGTTTCAAAATCTTCGATCATTTATCTTTC ACTGCGGAG	Amplification of a CRISPR-Cas9 plasmid pRCCN for deletion of *FAA2*, reverse
CC_FAA2-fw	GAAGATTTTGAAACCTTACGGTTTTAGAGCTAGAA ATAGCAAGTTAAAATAAGG	Amplification of a CRISPR-Cas9 plasmid pRCCN for deletion of *FAA2*, forward

The abbreviations within the primer names were used as follows: forward primer (fw) and reverse primer (rev)

### Fatty alcohol extraction

For the extraction of fatty alcohols from the culture medium, the cells were separated from the medium (8000 rcf, 3 min). 500 µL supernatant was mixed with 1 mL ethyl acetate containing 50 mg L^−1^ heptanol as internal standard and thoroughly shaken. After centrifugation (5000 rcf, 2 min), 500 µL of the organic phase was transferred to a gas chromatography (GC) vial. For the determination of the amount of fatty alcohols in the dodecane layer, 100 µL of the dodecane overlay was mixed with 900 µL ethyl acetate containing 50 mg L^−1^ heptanol as internal standard in a GC vial.

### Fatty acid extraction and derivatization

For the extraction of free fatty acids present in the culture medium, the cells were separated from the medium (3500 rcf, 10 min). An internal standard (0.2 mg heptanoic acid) was added to 10 mL supernatant and mixed with 1 mL 1 M HCl and 2.5 mL methanol–chloroform solution (1:1). The solution was vigorously shaken (5 min) and then centrifuged for 10 min at 3.000 rcf. The chloroform layer was recovered and evaporated overnight. The methylation of the fatty acids was performed as described in Ichihara and Fukubayashi [69]. The samples were dissolved in 200 µL of toluene, mixed with 1.5 mL of methanol and 300 µL of 8.0% (w/v) HCl solution [conc. HCl (35% w/w; 9.7 mL) was diluted with 41.5 mL of methanol], vortexed vigorously, and incubated at 100 °C for 3 h to form fatty acid methyl ester (FAME). After cooling under ice for 10 min, 1 mL H_2_O and 1 mL hexane were added to the sample. The mixture was shaken thoroughly, and the organic phase was transferred to a GC vial.

For the derivatization of the fatty acids from the in situ extraction experiment, the derivative reagent Bis(trimethylsilyl)-trifluoroacetamide (BSTFA) was used. After separation of the cells from the medium (3500 rcf, 10 min), 50 µL 1 M HCl and 0.01 mg heptanoic acid as an internal standard were added to 500 µL supernatant and mixed with 1 mL ethyl acetate. The ethyl acetate layer was evaporated overnight, and the samples were dissolved in 100 µL ethyl acetate and mixed with 100 µL BSTFA. For the determination of the amount of fatty acids in the dodecane layer, 20 µL of the dodecane layer was mixed with 80 µL ethyl acetate, 0.01 mg of heptanoic acid as an internal standard, and 100 µL BSTFA. The derivatization was done for 45 min at 80 °C. After cooling at 4 °C for 15 min, the samples were analyzed by GC.

### GC-FID analysis of FAMEs and fatty alcohols

The gas chromatographic measurements were carried out on a Perkin Elmer Clarus 400 system (Perkin Elmer, Germany) equipped with an Elite FFAP capillary column (30 m × 0.25 mm, film thickness: 0.25 µm; PerkinElmer, Germany) and a flame ionization detector (Perkin Elmer, Germany). 1 μL of the sample was analyzed after split injection (1:10); helium was used as carrier gas (90 kPa). For fatty acid methyl ester (FAME) quantification, the temperatures of the injector and detector were 200 and 250 °C, respectively. The following temperature program was applied: heating to 50 °C for 5 min; increase of 10 °C min^−1^ to 120 °C and hold for 5 min; increase at the rate of 15 °C min^−1^ to 180 °C and hold for 10 min; increase at the rate of 20 °C min^−1^ to 220 °C, and hold for 7 min. FAME were identified and quantified by comparison with authentic standard substances. For fatty alcohol quantification, an initial temperature of 50 °C was maintained for 5 min, followed by an increase at the rate of 20 °C min^−1^ to 210 °C and kept constant for 5 min. After a further increase at the rate of 20 °C min^−1^ to 230 °C, the temperature was kept constant for 6 min. The temperatures of both the injector and detector were 250 °C. Fatty alcohols were identified and quantified by comparison with authentic standard substances. For the in situ extraction experiment with dodecane as second phase, the column Elite 5MS capillary column (30 m × 0.25 mm, film thickness 1.00 µm, Perkin Elmer, Germany) was used for analysis of FAMEs and fatty alcohols. The temperatures of the injector and detector were 250 and 300 °C, respectively. The following temperature program was applied for FAMEs: 50 °C for 5 min, increase at the rate of 10 °C min^−1^ to 120 °C and hold for 5 min; increase at the rate of 15 °C min^−1^ to 220 °C, and hold for 10 min, increase at the rate of 20 °C min^−1^ to 300 °C, and hold for 5 min. For fatty alcohols, the following temperature program was applied: 50 °C for 5 min, increase at the rate of 20 °C min^−1^ to 220 °C and hold for 2 min; increase at the rate of 20 °C min^−1^ to 300 °C, and hold for 5 min.

## Abbreviations

OD_600_: optical density at 600 nm
PGK1: 3-Phosphoglycerate kinase
HXT7: high-affinity glucose transporter
PGM2: phosphoglucomutase
CYC1: cytochrome c
FBA1: fructose-1,6-bisphosphate aldolase
FAS: fatty acid synthase
FAA2: medium-chain fatty acyl-CoA synthetase
Ahr: aldehyde reductase
CAR: carboxylic acid reductase
ALR: aldehyde reductase
ADH: alcohol dehydrogenase
Sfp: phosphopantetheinyl transferase

## References

[1] Chapman L (2007). Transport and climate change: a review. J Transp Geogr..

[2] Edenhofer O, Pichs-Madruga R, Sokona Y, Farahani E, Kadner S, Seyboth K, Adler A, Baum I, Brunner S, Eickemeier P, Kriemann B, Savolainen J, Schlömer S, von Stechow C, Zwickel T, Minx JC, Contribution of Working Group III to the Fifth Assessment Report of the Intergovernmental Panel on Climate Change 2014 (2014). Climate Change. Mitigation of climate change.

[3] Peralta-Yahya PP, Zhang F, del Cardayre SB, Keasling JD (2012). Microbial engineering for the production of advanced biofuels. Nature.

[4] Karatzos S, van Dyk JS, McMillan JD, Saddler J. Drop-in biofuel production via conventional (lipid/fatty acid) and advanced (biomass) routes. Part I. Biofuels Bioprod Biorefining. 2017; 10.1002/bbb.1746.

[5] Junne S, Kabisch J (2017). Fueling the future with biomass: processes and pathways for a sustainable supply of hydrocarbon fuels and biogas. Eng Life Sci.

[6] Yu A-Q, Pratomo Juwono NK, Leong SSJ, Chang MW (2014). Production of fatty acid-derived valuable chemicals in synthetic microbes. Front Bioeng Biotechnol..

[7] Kang M-K, Nielsen J (2017). Biobased production of alkanes and alkenes through metabolic engineering of microorganisms. J Ind Microbiol Biotechnol.

[8] Cheon S, Kim HM, Gustavsson M, Lee SY (2016). Recent trends in metabolic engineering of microorganisms for the production of advanced biofuels. Curr Opin Chem Biol.

[9] Zargar A, Bailey CB, Haushalter RW, Eiben CB, Katz L, Keasling JD (2017). Leveraging microbial biosynthetic pathways for the generation of ‘drop-in’ biofuels. Curr Opin Biotechnol.

[10] Kremer F, Blank LM, Jones PR, Akhtar MK (2015). A comparison of the microbial production and combustion characteristics of three alcohol biofuels: ethanol, 1-butanol, and 1-octanol. Front Bioeng Biotechnol..

[11] Kerschgens B, Cai L, Pitsch H, Heuser B, Pischinger S (2016). Di- *n*-buthylether, *n*-octanol, and *n*-octane as fuel candidates for diesel engine combustion. Combust Flame.

[12] Rajesh Kumar B, Saravanan S, Rana D, Nagendran A (2016). A comparative analysis on combustion and emissions of some next generation higher-alcohol/diesel blends in a direct-injection diesel engine. Energy Convers Manag.

[13] Cai L, Uygun Y, Togbé C, Pitsch H, Olivier H, Dagaut P, Sarathy SM (2015). An experimental and modeling study of *n*-octanol combustion. Proc Combust Inst.

[14] Akhtar MK, Dandapani H, Thiel K, Jones PR (2015). Microbial production of 1-octanol: a naturally excreted biofuel with diesel-like properties. Metab Eng Commun..

[15] Atsumi S, Hanai T, Liao JC (2008). Non-fermentative pathways for synthesis of branched-chain higher alcohols as biofuels. Nature.

[16] Zhang K, Sawaya MR, Eisenberg DS, Liao JC (2008). Expanding metabolism for biosynthesis of nonnatural alcohols. Proc Natl Acad Sci USA..

[17] Dekishima Y, Lan EI, Shen CR, Cho KM, Liao JC (2011). Extending carbon chain length of 1-butanol pathway for 1-hexanol synthesis from glucose by engineered *Escherichia coli*. J Am Chem Soc.

[18] Dellomonaco C, Clomburg JM, Miller EN, Gonzalez R (2011). Engineered reversal of the β-oxidation cycle for the synthesis of fuels and chemicals. Nature.

[19] Wahlen BD, Oswald WS, Seefeldt LC, Barney BM (2009). Purification, characterization, and potential bacterial wax production role of an NADPH-dependent fatty aldehyde reductase from Marinobacter aquaeolei VT8. Appl Environ Microbiol.

[20] Willis RM, Wahlen BD, Seefeldt LC, Barney BM (2011). Characterization of a fatty acyl-CoA reductase from Marinobacter aquaeolei VT8: a bacterial enzyme catalyzing the reduction of fatty acyl-CoA to fatty alcohol. Biochemistry.

[21] Rowland O, Domergue F (2012). Plant fatty acyl reductases: enzymes generating fatty alcohols for protective layers with potential for industrial applications. Plant Sci.

[22] d’Espaux L, Ghosh A, Runguphan W, Wehrs M, Xu F, Konzock O (2017). Engineering high-level production of fatty alcohols by *Saccharomyces cerevisiae* from lignocellulosic feedstocks. Metab Eng.

[23] Haushalter RW, Groff D, Deutsch S, The L, Chavkin TA, Brunner SF (2015). Development of an orthogonal fatty acid biosynthesis system in *E. coli* for oleochemical production. Metab Eng.

[24] Runguphan W, Keasling JD (2014). Metabolic engineering of *Saccharomyces cerevisiae* for production of fatty acid-derived biofuels and chemicals. Metab Eng.

[25] Fillet S, Gibert J, Suárez B, Lara A, Ronchel C, Adrio JL (2015). Fatty alcohols production by oleaginous yeast. J Ind Microbiol Biotechnol.

[26] Feng X, Lian J, Zhao H (2015). Metabolic engineering of *Saccharomyces cerevisiae* to improve 1-hexadecanol production. Metab Eng.

[27] Hofvander P, Doan TTP, Hamberg M (2011). A prokaryotic acyl-CoA reductase performing reduction of fatty acyl-CoA to fatty alcohol. FEBS Lett.

[28] Foo JL, Susanto AV, Keasling JD, Leong SSJ, Chang MW (2017). Whole-cell biocatalytic and de novo production of alkanes from free fatty acids in *Saccharomyces cerevisiae*. Biotechnol Bioeng.

[29] Tang X, Feng L, Chen L, Chen WN (2017). Engineering *Saccharomyces cerevisiae* for efficient biosynthesis of fatty alcohols based on enhanced supply of free fatty acids. ACS Omega..

[30] Zhou YJ, Buijs NA, Zhu Z, Qin J, Siewers V, Nielsen J (2016). Production of fatty acid-derived oleochemicals and biofuels by synthetic yeast cell factories. Nat Commun..

[31] Akhtar MK, Turner NJ, Jones PR (2013). Carboxylic acid reductase is a versatile enzyme for the conversion of fatty acids into fuels and chemical commodities. Proc Natl Acad Sci USA..

[32] Venkitasubramanian P, Daniels L, Rosazza JPN (2007). Reduction of carboxylic acids by Nocardia aldehyde oxidoreductase requires a phosphopantetheinylated enzyme. J Biol Chem.

[33] Buijs NA, Zhou YJ, Siewers V, Nielsen J (2015). Long-chain alkane production by the yeast *Saccharomyces cerevisiae*. Biotechnol Bioeng.

[34] Wang M, Wu H, Xu J, Li C, Wang Y, Wang Z (2017). Five fatty acyl-coenzyme A reductases are involved in the biosynthesis of primary alcohols in *Aegilops tauschii* leaves. Front Plant Sci..

[35] Steen EJ, Kang Y, Bokinsky G, Hu Z, Schirmer A, McClure A (2010). Microbial production of fatty-acid-derived fuels and chemicals from plant biomass. Nature.

[36] Moura M, Pertusi D, Lenzini S, Bhan N, Broadbelt LJ, Tyo KEJ (2016). Characterizing and predicting carboxylic acid reductase activity for diversifying bioaldehyde production. Biotechnol Bioeng.

[37] Finnigan W, Thomas A, Cromar H, Gough B, Snajdrova R, Adams JP (2017). Characterization of carboxylic acid reductases as enzymes in the toolbox for synthetic chemistry. ChemCatChem..

[38] Wang G, Xiong X, Ghogare R, Wang P, Meng Y, Chen S (2016). Exploring fatty alcohol-producing capability of *Yarrowia lipolytica*. Biotechnol Biofuels.

[39] Marcheschi RJ, Li H, Zhang K, Noey EL, Kim S, Chaubey A (2012). A synthetic recursive “+1” pathway for carbon chain elongation. ACS Chem Biol.

[40] Machado HB, Dekishima Y, Luo H, Lan EI, Liao JC (2012). A selection platform for carbon chain elongation using the CoA-dependent pathway to produce linear higher alcohols. Metab Eng.

[41] Sheng J, Stevens J, Feng X (2016). Pathway Compartmentalization in peroxisome of *Saccharomyces cerevisiae* to produce versatile medium chain fatty alcohols. Sci Rep..

[42] Gibson BR, Lawrence SJ, Leclaire JPR, Powell CD, Smart KA (2007). Yeast responses to stresses associated with industrial brewery handling. FEMS Microbiol Rev.

[43] Weber C, Farwick A, Benisch F, Brat D, Dietz H, Subtil T, Boles E (2010). Trends and challenges in the microbial production of lignocellulosic bioalcohol fuels. Appl Microbiol Biotechnol.

[44] Li M, Borodina I (2015). Application of synthetic biology for production of chemicals in yeast *Saccharomyces cerevisiae*. FEMS Yeast Res.

[45] Fernandez-Moya R, Da Silva NA (2017). Engineering *Saccharomyces cerevisiae* for high-level synthesis of fatty acids and derived products. FEMS Yeast Res.

[46] Fischer M, Rhinow D, Zhu Z, Mills DJ, Zhao ZK, Vonck J, Grininger M (2015). Cryo-EM structure of fatty acid synthase (FAS) from *Rhodosporidium toruloides* provides insights into the evolutionary development of fungal FAS. Protein Sci.

[47] Jenni S, Leibundgut M, Boehringer D, Frick C, Mikolásek B, Ban N (2007). Structure of fungal fatty acid synthase and implications for iterative substrate shuttling. Science.

[48] Johansson P, Mulinacci B, Koestler C, Vollrath R, Oesterhelt D, Grininger M (2009). Multimeric options for the auto-activation of the *Saccharomyces cerevisiae* FAS type I megasynthase. Structure..

[49] Leibundgut M, Jenni S, Frick C, Ban N (2007). Structural basis for substrate delivery by acyl carrier protein in the yeast fatty acid synthase. Science.

[50] Lomakin IB, Xiong Y, Steitz TA (2007). The crystal structure of yeast fatty acid synthase, a cellular machine with eight active sites working together. Cell.

[51] Leber C, Da Silva NA (2014). Engineering of *Saccharomyces cerevisiae* for the synthesis of short chain fatty acids. Biotechnol Bioeng.

[52] Zhu Z, Zhou YJ, Krivoruchko A, Grininger M, Zhao ZK, Nielsen J (2017). Expanding the product portfolio of fungal type I fatty acid synthases. Nat Chem Biol.

[53] Gajewski J, Pavlovic R, Fischer M, Boles E, Grininger M (2017). Engineering fungal de novo fatty acid synthesis for short chain fatty acid production. Nat Commun..

[54] Gajewski J, Buelens F, Serdjukow S, Janßen M, Cortina N, Grubmüller H, Grininger M (2017). Engineering fatty acid synthases for directed polyketide production. Nat Chem Biol.

[55] Xu P, Qiao K, Ahn WS, Stephanopoulos G (2016). Engineering *Yarrowia lipolytica* as a platform for synthesis of drop-in transportation fuels and oleochemicals. Proc Natl Acad Sci USA..

[56] Rigouin C, Gueroult M, Croux C, Dubois G, Borsenberger V, Barbe S (2017). Production of medium chain fatty acids by *Yarrowia lipolytica*: combining molecular design and TALEN to engineer the fatty acid synthase. ACS Synth Biol..

[57] van Roermund CWT, Ijlst L, Majczak W, Waterham HR, Folkerts H, Wanders RJA, Hellingwerf KJ (2012). Peroxisomal fatty acid uptake mechanism in *Saccharomyces cerevisiae*. J Biol Chem.

[58] Leber C, Choi JW, Polson B, Da Silva NA (2016). Disrupted short chain specific β-oxidation and improved synthase expression increase synthesis of short chain fatty acids in *Saccharomyces cerevisiae*. Biotechnol Bioeng.

[59] Schonauer MS, Kastaniotis AJ, Kursu VAS, Hiltunen JK, Dieckmann CL (2009). Lipoic acid synthesis and attachment in yeast mitochondria. J Biol Chem.

[60] Borrull A, López-Martínez G, Poblet M, Cordero-Otero R, Rozès N (2015). New insights into the toxicity mechanism of octanoic and decanoic acids on *Saccharomyces cerevisiae*. Yeast.

[61] Asadollahi MA, Maury J, Møller K, Nielsen KF, Schalk M, Clark A, Nielsen J (2008). Production of plant sesquiterpenes in *Saccharomyces cerevisiae*: effect of ERG9 repression on sesquiterpene biosynthesis. Biotechnol Bioeng.

[62] Qun J, Shanjing Y, Lehe M (2002). Tolerance of immobilized baker’s yeast in organic solvents. Enzyme Microb Technol..

[63] Generoso WC, Gottardi M, Oreb M, Boles E (2016). Simplified CRISPR-Cas genome editing for *Saccharomyces cerevisiae*. J Microbiol Methods.

[64] Bruder S, Reifenrath M, Thomik T, Boles E, Herzog K (2016). Parallelised online biomass monitoring in shake flasks enables efficient strain and carbon source dependent growth characterisation of *Saccharomyces cerevisiae*. Microb Cell Fact.

[65] Gietz RD, Schiestl RH (2007). Frozen competent yeast cells that can be transformed with high efficiency using the LiAc/SS carrier DNA/PEG method. Nat Protoc.

[66] Wiedemann B, Boles E (2008). Codon-optimized bacterial genes improve l-Arabinose fermentation in recombinant *Saccharomyces cerevisiae*. Appl Environ Microbiol.

[67] Gibson DG, Young L, Chuang R-Y, Venter JC, Hutchison CA, Smith HO (2009). Enzymatic assembly of DNA molecules up to several hundred kilobases. Nat Methods.

[68] Farwick A, Bruder S, Schadeweg V, Oreb M, Boles E (2014). Engineering of yeast hexose transporters to transport d-xylose without inhibition by d-glucose. Proc Natl Acad Sci USA..

[69] Ichihara KI, Fukubayashi Y (2010). Preparation of fatty acid methyl esters for gas-liquid chromatography. J Lipid Res.

